# Krüppel-homolog 1 exerts anti-metamorphic and vitellogenic functions in insects via phosphorylation-mediated recruitment of specific cofactors

**DOI:** 10.1186/s12915-021-01157-3

**Published:** 2021-10-08

**Authors:** Zhongxia Wu, Libin Yang, Huihui Li, Shutang Zhou

**Affiliations:** State Key Laboratory of Cotton Biology, Key Laboratory of Plant Stress Biology, School of Life Sciences, Henan University, Kaifeng, 475004 China

**Keywords:** Kr-h1, Juvenile hormone, E93, Insect metamorphosis, Female reproduction

## Abstract

**Background:**

The zinc-finger transcription factor Krüppel-homolog 1 (Kr-h1) exerts a dual regulatory role during insect development by preventing precocious larval/nymphal metamorphosis and in stimulating aspects of adult reproduction such as vitellogenesis. However, how Kr-h1 functions both as a transcriptional repressor in juvenile metamorphosis and an activator in adult reproduction remains elusive. Here, we use the insect *Locusta migratoria* to dissect the molecular mechanism by which Kr-h1 functions as activator and repressor at these distinct developmental stages.

**Results:**

We report that the kinase PKCα triggers Kr-h1 phosphorylation at the amino acid residue Ser^154^, a step essential for its dual functions. During juvenile stage, phosphorylated Kr-h1 recruits a corepressor, C-terminal binding protein (CtBP). The complex of phosphorylated Kr-h1 and CtBP represses the transcription of *Ecdysone induced protein 93F* (*E93*) and consequently prevents the juvenile-to-adult transition. In adult insects, phosphorylated Kr-h1 recruits a coactivator, CREB-binding protein (CBP), and promotes vitellogenesis by inducing the expression of *Ribosomal protein L36*. Furthermore, Kr-h1 phosphorylation with the concomitant inhibition of *E93* transcription is evolutionarily conserved across insect orders.

**Conclusion:**

Our results suggest that Kr-h1 phosphorylation is indispensable for the recruitment of transcriptional cofactors, and for its anti-metamorphic and vitellogenic actions in insects. Our data shed new light on the understanding of Kr-h1 regulation and function in JH-regulated insect metamorphosis and reproduction.

**Supplementary Information:**

The online version contains supplementary material available at 10.1186/s12915-021-01157-3.

## Background

Juvenile hormone (JH), an arthropod-specific sesquiterpenoid secreted by the corpora allata, plays a central role in insect metamorphosis and reproduction. In juvenile stages, JH maintains the larval/nymphal status by suppressing the metamorphic action of the steroid hormone 20-hydroxyecdysone (20E) [1–5]. In adult insects, JH stimulates aspects of reproduction including post-emergence development, vitellogenesis, and oogenesis [6, 7]. *Krüppel-homolog 1* (*Kr-h1*) is a primary JH early-inducible gene coding for a zinc-finger transcription factor that mediates both anti-metamorphic and vitellogenic actions of JH [8–11]. Kr-h1 prevents immature larvae from precocious larval-pupal metamorphosis by inhibiting the transcription of pupa-specifier gene *Broad-complex* (*Br-C*) in holometabolous insects [12–14]. Kr-h1 also prevents precocious nymphal-adult or pupal-adult transition by inhibiting the expression of *Ecdysone induced protein 93F* (*E93*), an adult-specifier gene in both hemimetabolous and holometabolous insects [14–17] in the context of the MEKRE93 pathway, the general regulatory axis of insect metamorphosis [8, 15]. In addition, Kr-h1 suppresses 20E biosynthesis by inhibiting the expression of steroidogenic enzyme gene *Spok* in prothoracic glands of the fruit fly *Drosophila melanogaster* and thus prevents precocious larval-pupal transformation [18]. Stimulation of female reproduction by Kr-h1 is reported in a variety of insect species [9, 10, 19]. RNAi-mediated knockdown of *Kr-h1* resulted in blocked vitellogenesis and impaired egg development in the migratory locust *Locusta migratoria*, the rice borer *Chilo suppressalis*, the oriental fruit fly *Bactrocera dorsalis*, the cotton bollworm *Helicoverpa armigera*, and the brown planthopper *Nilaparvata lugens* [20–24]. In the mosquito *Aedes aegypti*, Kr-h1 regulates the developmental phase in preparation for competence acquisition for blood feeding, as well as subsequent vitellogenesis and egg development [25–27]. In the common bed bug *Cimex lectularius*, depletion of *Kr-h1* in adult females caused severely reduced egg hatchability [28].

*Kr-h1* is transcriptionally activated by the JH-receptor complex comprising Methoprene-tolerant (Met) and Taiman, two members of the bHLH-PAS transcription factor family [15, 29–33]. Met also dimerizes with Cycle, which upregulates *Kr-h1* transcription in JH-mediated previtellogenic development of *Ae. aegypti* [25]. In the beetle *Tribolium castaneum*, JH represses the expression of *Histone deacetylase 1* (*HDAC1*), leading to increased levels of histone acetylation and consequently promoting *Kr-h1* transcription [34, 35]. Beside transcriptional regulation, *Kr-h1* is post-transcriptionally regulated by microRNAs [19, 36]. In the cockroach *Blattella germanica*, miR-2 eliminates *Kr-h1* transcripts at final instar nymphs, which crucially contributes to the onset of metamorphosis [37]. In *L. migratoria*, *Kr-h1* is downregulated by let-7 and miR-278, whereas JH suppresses the expression of these two miRNAs. This regulatory loop ensures a proper level of Kr-h1 essential for preventing precocious metamorphosis in nymphs and stimulating JH-dependent vitellogenesis in adults [38–44].

In an effort to elucidate how Kr-h1 functions in repressing precocious nymph metamorphosis and stimulating adult reproduction in *L. migratoria*, we investigated Kr-h1 phosphorylation and its involvement in transcriptional repression and activation. The migratory locust *L. migratoria* is a destructive insect pest worldwide as well as a representative of evolutionarily basal insects with hemimetabolous development and JH-dependent vitellogenesis. We found that PKCα triggers Kr-h1 phosphorylation. Phosphorylated Kr-h1 recruited C-terminal binding protein (CtBP), consequently inhibiting *E93* expression and nymphal-adult metamorphosis. Phosphorylated Kr-h1 interacted with CREB-binding protein (CBP), which stimulated the transcription of *Ribosomal protein L36* (RL36) and reproduction. We also provide evidence that the essential role of phosphorylated Kr-h1 in recruiting CtBP and repressing *E93* expression is evolutionarily conserved in other representative insects including the silkworm *Bombyx mori*, the beetle *T. castaneum* and the fruit fly *D. melanogaster*.

## Results

### Kr-h1 is phosphorylated by PKCα at Ser^154^

**Fig. 1:**
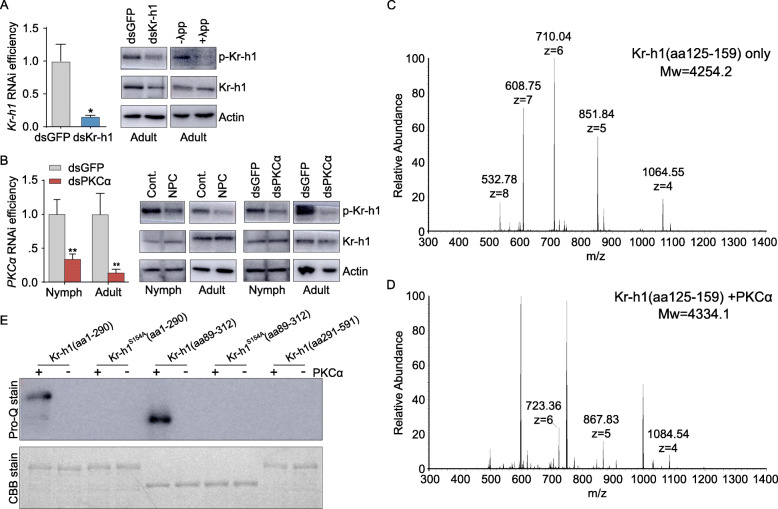
Phosphorylation of Kr-h1 by PKCα at Ser^154^. **A** Left panel: *Kr-h1* RNAi efficiency in the fat body of 3-day-old adult females. *P<0.05. *n*=8. Right panel: Verification of phospho-Kr-h1 (Ser^154^) antibody specificity by using protein extracts from the fat body of 3-day-old adult females subjected to *Kr-h1* knockdown and phosphatase λpp treatment. **B** Left panel: *PKCα* RNAi efficiency in the whole body of penultimate 4th instar nymphs and the fat body of 3-day-old adult females. **P<0.01. *n*=8. Right panel: Relative levels of Kr-h1 and phosphorylated Kr-h1 (p-Kr-h1) in the whole body of 4th instar nymphs and the fat body of 3-day-old adult females treated by NPC15437 (NPC) vs. DMSO solvent control (Cont.) and dsPKCα vs. dsGFP control. **C-D** LC-MS/MS analysis of wildtype Kr-h1(aa125-159) peptide (**C**) and Kr-h1(aa125-159) preincubated with PKCα (**D**). m/z indicates the mass to charge ratio. **E** Upper panel: Pro-Q Diamond Phosphoprotein Gel Stain of purified bacterially-expressed GST-tagged peptides of Kr-h1(aa1-290), Kr-h1^S154A^(aa1-290), Kr-h1(aa89-312), Kr-h1^S154A^(aa89-312), and Kr-h1(aa291-591) preincubated with or without PKCα. Lower panel: Coomassie brilliant blue staining was used as the loading controls

We initially predicted the phosphorylation of *L. migratoria* Kr-h1 (GenBank: KJ425482) computationally by DISPHOS (V1.3) software [45]. Three serine residues, Ser^154^, Ser^371^, and Ser^554^ were suggested as potential phosphorylation sites, with Ser^154^ at the highest score (Additional file 1: Fig. S1A). To validate Kr-h1 phosphorylation, we performed immunoprecipitation using a commercial anti-phospho-(Ser) antibody and a polyclonal anti-Kr-h1 antibody [38]. Phosphorylated Kr-h1 (p-Kr-h1) was detected in protein extracts from both nymphs and adults (Additional file 1: Fig. S1B). We generated an anti-phospho-Kr-h1 (Ser^154^) antibody (Additional file 1: Fig. S1C). Its specificity was verified by western blot using proteins extracted from adult female fat bodies subjected to *Kr-h1* knockdown as well as those treated with phosphatase λpp (Fig. 1A). The specificity of anti-phospho-Kr-h1 (Ser^154^) antibody was also verified by western blot using the recombinant Flag-tagged proteins of wildtype Kr-h1 and mutated Kr-h1^S154A^(Ser^154^ to Ala^154^) expressed in *Drosophila* S2 cells treated with methoprene as well as the bacterially expressed GST-tagged peptides of Kr-h1(aa1-290) and Kr-h1^S154A^(aa1-290) incubated with PKCα (Additional file 1: Fig. S1D). We next investigated the kinase triggering Kr-h1 phosphorylation at Ser^154^. The motif KAFSVK at amino acid residues 151-156 of *L. migratoria* Kr-h1 (Additional file 1: Fig. S1A) is a conserved motif recognized by PKC [46–48], presumably PKCα and PKCη as predicted by a GPS algorithm [49]. As evaluated by western blots, application of the PKC inhibitor NPC15437 in nymphs and adult females reduced p-Kr-h1 levels (Fig. 1B). Depletion of *PKCα* (GenBank: MT081310) in nymphs and adult females caused significant reduction of p-Kr-h1 but not total Kr-h1 abundance (Fig. 1B and Additional file 1: Fig. S2A). In contrast, *PKCη* (GenBank: MT081311) knockdown had no obvious effect on Kr-h1 phosphorylation (Additional file 1: Fig. S2B). These results imply that PKCα is likely to mediate Kr-h1 phosphorylation at Ser^154^. To confirm the action of PKCα on Kr-h1 phosphorylation, we synthesized wildtype Kr-h1(aa125-159) and mutated Kr-h1^S154A^(aa125-159) peptides, followed by incubating them separately with PKCα for LC-MS/MS analysis. As illustrated in Fig. 1C, Kr-h1(aa125-159) peptide without PKCα treatment had a molecular mass of 4,254 Da. However, incubation of Kr-h1(aa125-159) peptide with PKCα yielded a molecular mass of 4,334 Da (Fig. 1D), exhibiting an 80 Da shift compared to Kr-h1(aa125-159) peptide without PKCα treatment. When mutated Kr-h1^S154A^(aa125-159) peptide was incubated with PKCα, a molecular mass of 4238 Da was detected, same as that observed with Kr-h1^S154A^(aa125-159) peptide alone (Additional file 1: Fig. S2C). To further define PKCα-mediated Kr-h1 phosphorylation at Ser^154^, we carried out Pro-Q Diamond Phosphoprotein Gel Staining with purified bacterially expressed GST-tagged peptides of Kr-h1(aa1-290), Kr-h1^S154A^(aa1-290), Kr-h1(aa89-312), Kr-h1^S154A^(aa89-312), and Kr-h1(aa291-591) incubated with PKCα. As shown in Fig. 1E, the specific phosphorylation bands were observed with wildtype Kr-h1(aa1-290) and Kr-h1(aa89-312) peptides, but not mutated Kr-h1^S154A^(aa1-290) or Kr-h1^S154A^(aa89-312). No phosphorylation band was observed with the truncated Kr-h1(aa291-591) (Fig. 1E), indicating that PKCα-mediated Kr-h1 phosphorylation is unlikely to occur at Ser^371^ or Ser^554^.

### Kr-h1 expression and phosphorylation are in response to JH

**Fig. 2:**
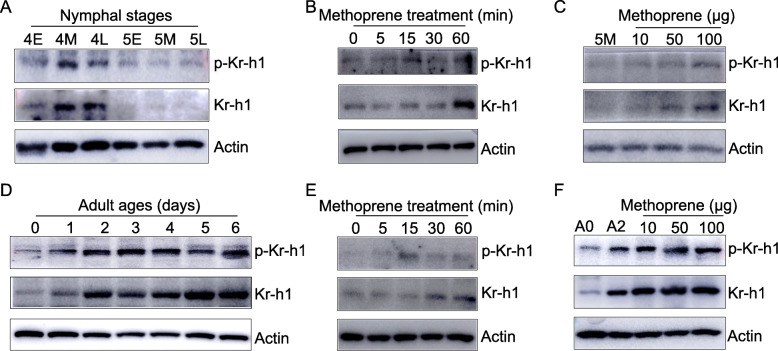
Responsiveness of Kr-h1 phosphorylation to JH. **A** Abundance of Kr-h1 and p-Kr-h1 in the whole body of penultimate 4th and final 5th instar nymphs. E, M and L indicate the early (day 1), mid (day 2 for 4th, and day 3 for 5th), and late (day 4 for 4th, and day 5 for 5th) stages, respectively. **B** Relative levels of Kr-h1 and p-Kr-h1 in mid-5th instar nymphs and in those further treated with methoprene at 100 μg per locust for 5–60 min. **C** Relative abundance of Kr-h1 and p-Kr-h1 in mid-5th instar nymphs (5M) and those further treated with methoprene at 10–100 μg per locust for 8 h. **D** Developmental dynamics of Kr-h1 and p-Kr-h1 in the fat body of adult females at 0–6 days post adult emergence. **E** Relative levels of Kr-h1 and p-Kr-h1 in the fat body of newly-emerged adult females (A0) as well as those further treated with methoprene at 100 μg per locust for 5–60 min. **F** Relative levels of Kr-h1 and p-Kr-h1 in the fat body of newly-emerged adult females (A0) as well as those further treated with methoprene at 10–100 μg per locust for 8 h. A2, 2-day-old adult female as a control

To explore the dynamics of p-Kr-h1 before the onset of locust metamorphosis, we conducted western blot using proteins extracted from the penultimate 4th and final 5th instar nymphs. As shown in Fig. 2A, p-Kr-h1 levels were high in mid and late 4th instar nymphs but markedly declined in 5th instar nymphs. The decreased levels of p-Kr-h1 at final nymphal instar appeared to correlate with the decline of JH titer in this phase [50], suggesting a possible effect of JH on Kr-h1 phosphorylation. It should be noted that *Kr-h1* is expressed in response to JH [21, 38]. The abundance of total Kr-h1 also decreased in 5th instar nymphs (Fig. 2A). To evaluate the responsiveness of Kr-h1 phosphorylation to JH in juvenile stage, western blot was performed using protein extracts from mid-5th instar nymphs as well as those further treated with methoprene for 5-60 min. Application of methoprene caused increase of both Kr-h1 and p-Kr-h1 levels, and longer exposure to methoprene tended to have a relatively more pronounced effect on Kr-h1 expression and phosphorylation (Fig. 2B). Notably, p-Kr-h1 levels increased more rapidly than total Kr-h1 after 15-min exposure to methoprene (Fig. 2B and Additional file 1: Fig. S3), implying a role of JH in stimulating Kr-h1 phosphorylation. Dose-response experiments demonstrated that higher doses of methoprene induced higher levels of Kr-h1 and p-Kr-h1 (Fig. 2C). The data suggest that JH promotes Kr-h1 expression and phosphorylation in nymphs, and the high levels of Kr-h1 phosphorylation are generally observed with more abundant Kr-h1 proteins.

We next studied the temporal abundance of p-Kr-h1 after adult ecdysis using protein extracts from the fat body of adult females at 0–6 days post adult emergence (PAE). Compared to that on the day of adult emergence, p-Kr-h1 levels increased at 1–4 days PAE and remained high on days 5–6, resembling that of total Kr-h1 (Fig. 2D). As JH is undetectable in the hemolymph at adult emergence but sharply increases thereafter [51], the enhanced levels of Kr-h1 and p-Kr-h1 appeared to positively correlate with elevated hemolymph JH titer. To elucidate the responsiveness of Kr-h1 phosphorylation to JH in adult locusts, western blot analysis was carried out using protein extracts isolated from the fat body of newly emerged adult females and those further treated with methoprene. As observed in nymphs, methoprene-induced Kr-h1 expression and phosphorylation were also seen in adults (Fig. 2E, F). Likewise, p-Kr-h1 abundance increased more rapidly than total Kr-h1 in the fat body of adult females treated with methoprene for 15 min (Fig. 2E and Additional file 1: S3). Taken together, our data suggest that JH-induced Kr-h1 expression is accompanied by increased levels of Kr-h1 phosphorylation in both nymphal and adult locusts.

### Kr-h1 phosphorylation is required for its anti-metamorphic action

**Fig. 3:**
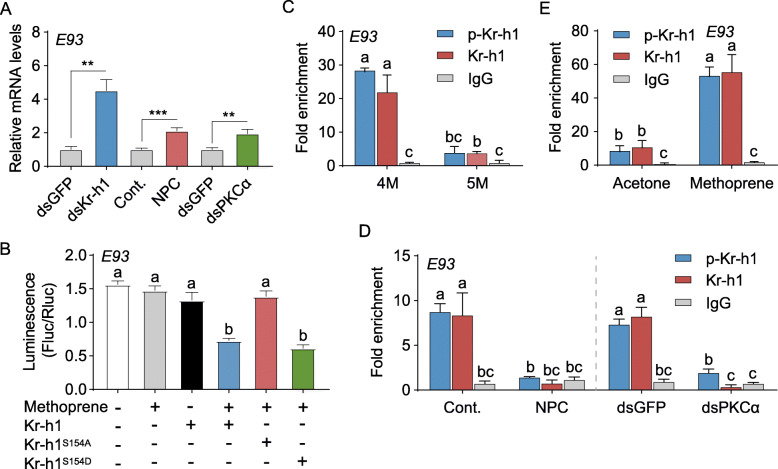
Requirement of Kr-h1 phosphorylation in inhibiting *E93* transcription. **A** Relative levels of *E93* mRNA in mid-4th instar nymphs treated with dsKr-h1 vs. dsGFP control, NPC15437 (NPC) vs. DMSO solvent control (Cont.), and dsPKCα vs. dsGFP control. **P<0.01 and ***P<0.001. *n*=8. **B** Luciferase reporter assays using S2 cells co-transfected with pGL4.10-4×E93^-623 to -606^ plus pAc5.1/Flag-Kr-h1, pAc5.1/Flag-Kr-h1^S154A^, pAc5.1/Flag-Kr-h1^S154D^, or pAc5.1/Flag empty control with or without 10 μM methoprene treatment. Co-transfection of pGL4.10-4×E93^-623 to -606^ and pAc5.1/Flag empty control without methoprene treatment was used as the control. Means labeled with different letters indicate significant difference at *P*<0.05. *n*=4. **C** ChIP assays showing relative precipitation of *E93* promoter region with the KBS motif (RPEP-KBS) in mid-4th (4M) and 5th (5M) instar nymphs. **D** RPEP-KBS in 4M nymphs treated with NPC15437 (NPC) vs. DMSO solvent control (Cont.) and dsPKCα vs. dsGFP control. **E** RPEP-KBS in 5M nymphs treated with 50 μg methoprene vs. acetone solvent control. In **C**–**E**, p-Kr-h1, phospho-Kr-h1 (Ser^154^) antibody; Kr-h1, Kr-h1 antibody; and IgG, non-specific rabbit IgG control. Means labeled with different letters indicate significant difference at *P*<0.05. *n*=4

Previous studies have documented that E93 controls metamorphic nymphal-adult or pupal-adult transition [14–16]. Kr-h1 represses *E93* transcription [15] by binding to the promoter sequence bearing the core Kr-h1 binding site (KBS) [17]. As expected, depletion of *E93* (GenBank: MT081312) in the final instar nymph of locusts resulted in supernumerary nymphs and delayed adult morphogenesis (Additional file 1: Fig. S4). Knockdown of *Kr-h1* in penultimate instar nymphs caused 4.5-fold increase of *E93* transcripts (Fig. 3A). Application of a PKC inhibitor, NPC15437, or knockdown of *PKCα* led to significant increase of *E93* mRNA levels (Fig. 3A), suggesting the possible requirement of Kr-h1 phosphorylation for repressing *E93* transcription. Analysis of upstream 3-kb sequence revealed a conserved KBS in the proximal promoter region (nt -617 to -612) of *L. migratoria E93* gene (Additional file 1: Fig. S5A). We then carried out dual luciferase reporter assays by co-transfection of pGL4.10-4×E93^-623 to -606^ with pAc5.1/Flag-Kr-h1, pAc5.1/Flag-Kr-h1^S154A^, pAc5.1/Flag-Kr-h1^S154D^ or pAc5.1/Flag empty control into *Drosophila* S2 cells treated with methoprene. Western blot demonstrated that methoprene treatment stimulated Flag-Kr-h1 phosphorylation (Additional file 1: Fig. S5B). Overexpression of Flag-Kr-h1 plus methoprene treatment caused about 58% reduction of *E93* reporter activity compared to the empty vector control (Fig. 3B). The capacity of Kr-h1 to inhibit *E93* reporter activity was blocked by overexpression of Flag-Kr-h1^S154A^, a mutated p-Kr-h1 (Fig. 3B). In contrast, overexpression of p-Kr-h1 wildtype variant, Flag-Kr-h1^S154D^, restored the inhibitory constraints of Kr-h1 on *E93* reporter activity (Fig. 3B). As illustrated in Additional file 1: Fig. S5B, Flag-Kr-h1^S154D^ but not Flag-Kr-h1^S154A^ was recognized by the anti-phospho-Kr-h1 (Ser^154^) antibody. Knowing that p-Kr-h1 had an essential role in suppressing *E93* reporter activity, we next performed in vivo ChIP analysis using anti-phospho-Kr-h1 (Ser^154^) antibody and nuclear extracts from mid-4th and 5th instar nymphs. The antibodies against Kr-h1 and IgG were used as the positive and negative controls, respectively. As shown in Fig. 3C, p-Kr-h1 was remarkably enriched with *E93* promoter region covering the KBS motif in penultimate 4th instar nymphs in which JH, Kr-h1, and p-Kr-h1 were in high levels. Conversely, a marginal precipitation of p-Kr-h1 was observed at final 5th nymphal instar when JH, Kr-h1, and p-Kr-h1 levels were low (Fig. 3C). NPC15437 treatment or *PKCα* knockdown restrained p-Kr-h1 enrichment with KBS-containing *E93* promoter region in 4th instar nymphs (Fig. 3D). Moreover, methoprene treatment of 5th instar nymphs caused noticeable increase of p-Kr-h1 enrichment (Fig. 3E). Collectively, these results suggest an essential role of Kr-h1 phosphorylation in transcriptional repression of *E93* in the nymphs of *L. migratoria*.

### Kr-h1 phosphorylation is required for its role in stimulating reproduction

**Fig. 4:**
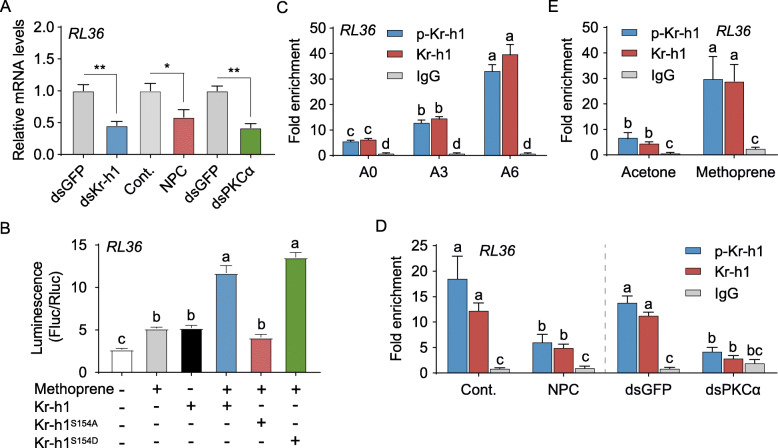
Requirement of Kr-h1 phosphorylation in induction of *RL36* transcription. **A** Relative levels of *RL36* transcript in the fat body of 3-day-old adult females treated with dsKr-h1 vs. dsGFP control, NPC15437 (NPC) vs. DMSO solvent control (Cont.), and dsPKCα vs. dsGFP control. *P<0.05 and **P<0.01. *n*=8. **B** Luciferase reporter assays using S2 cells co-transfected with pGL4.10-4×RL36^-1647 to -1632^ plus pAc5.1/Flag-Kr-h1, pAc5.1/Flag-Kr-h1^S154A^, pAc5.1/Flag-Kr-h1^S154D^, or pAc5.1/Flag empty control. Methoprene was applied at 10 μM. Co-transfection of pGL4.10-4×RL36^-1647 to -1632^ and pAc5.1/Flag empty vector without methoprene treatment was used as the control. Means labeled with different letters indicate significant difference at *P*<0.05. *n*=4. **C** ChIP assays showing relative precipitation of *RL36* promoter region with the KBS motif (RPRP-KBS) in the fat body of adult females on day 0 (A0), day 3 (A3), and day 6 (A6). **D** RPRP-KBS in the fat body of 3-day-old adult females treated with NPC15437 (NPC) vs. DMSO solvent control (Cont.) and dsPKCα vs. dsGFP control. **E** RPRP-KBS in the fat body of 3-day-old adult females treated with 50 μg methoprene vs. acetone solvent control. In **C**–**E**, p-Kr-h1, phospho-Kr-h1 (Ser^154^) antibody; Kr-h1, Kr-h1 antibody; and IgG, non-specific rabbit IgG control. Means labeled with different letters indicate significant difference at *P*<0.05. *n*=4

Kr-h1 has a dual role in preventing precocious nymphal/larval metamorphosis and in promoting adult reproduction. In *L. migratoria, Ribosomal protein L36 (RL36)* (GenBank: MT081313) was previously found to express in response to the JH-Met-Kr-h1 pathway [52]. RL36 is a component of the 60S subunit of ribosomes involved in ribosome biogenesis and protein translation as well as extra-ribosomal functions in various cellular processes [53]. Knocking down *RL36* resulted in blocked ovarian growth and arrested oocyte maturation (Additional file 1: Fig. S6). As shown in Fig. 4A, *Kr-h1* knockdown caused 54% reduction of *RL36* mRNA levels. Similarly, NPC15437 treatment and *PKCα* knockdown resulted in 41% and 58% decrease of *RL36* transcripts, respectively (Fig. 4A), suggesting a possible role of p-Kr-h1 in *RL36* expression. For luciferase reporter assay, *RL36* promoter region (nt -1647 to -1632) comprising a KBS motif (Additional file 1: Fig. S5A) was cloned into pGL4.10 vector. Co-transfection of pAc5.1/Flag-Kr-h1 and pGL4.10-4×RL36^-1647 to -1632^ in S2 cells treated with methoprene brought about 2-fold induction of *RL36* reporter activity compared to the empty vector control (Fig. 4B). When pAc5.1/Flag-Kr-h1^S154A^ was co-transfected with pGL4.10-4×RL36^-1647 to -1632^, no significant induction of *RL36* reporter activity was observed (Fig. 4B). However, the induction of *RL36* reporter activity was restored by overexpression of Flag-Kr-h1^S154D^ (Fig. 4B). The data indicate an essential role of Kr-h1 phosphorylation in *RL36* transcription. We next performed ChIP assays to quantify in vivo binding of p-Kr-h1 to KBS-containing promoter region of *RL36* in the fat body of adult females. Compared to the day of adult emergence, p-Kr-h1 was more enriched with the KBS-containing promoter sequence of *RL36* on day 3, and even more on day 6 (Fig. 4C). However, NPC15437 treatment and *PKCα* knockdown in 6-day-old adult females resulted in significant reduction of p-Kr-h1 enrichment with *RL36* promoter (Fig. 4D). Furthermore, application of methoprene to newly emerged adult females led to significantly enhanced precipitation of p-Kr-h1 in *RL36* promoter region (Fig. 4E). These results together indicate a pivotal role of Kr-h1 phosphorylation in induction of *RL36* transcription during female reproduction.

### Phosphorylated Kr-h1 recruits distinct cofactors in anti-metamorphic and vitellogenic actions

**Fig. 5:**
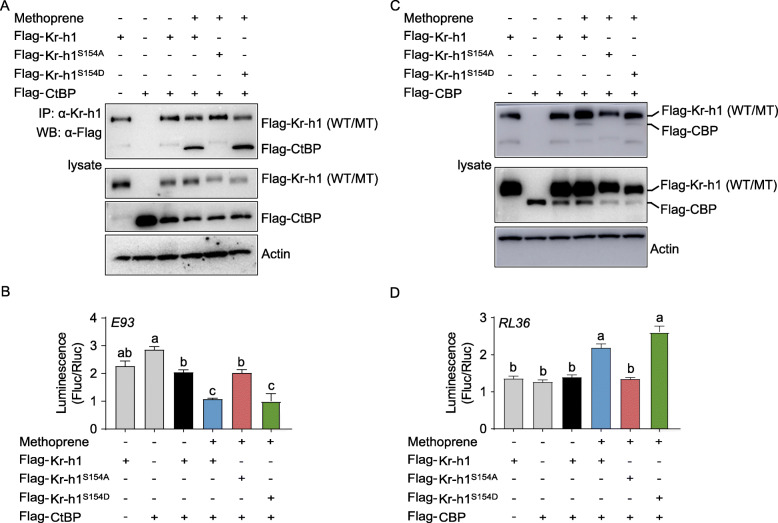
Essential role of Kr-h1 phosphorylation in the interaction with transcriptional cofactors. **A** Upper panel: immunoprecipitation (IP) and western blot (WB) showing the interaction of Flag-Kr-h1, Flag-Kr-h1^S154A^, or Flag-Kr-h1^S154D^ with Flag-CtBP. Middle and lower panels: the expression of above recombinant proteins in S2 cells. α-Kr-h1, Kr-h1 antibody; α-Flag, Flag antibody. WT, wildtype; MT, mutant. **B** Luciferase reporter assays after co-transfection of pGL4.10-4×E93^-623 to -606^ and pAc5.1/Flag-CtBP plus pAc5.1/Flag-Kr-h1, pAc5.1/Flag-Kr-h1^S14A^, or pAc5.1/Flag-Kr-h1^S154D^ into S2 cells. Co-transfection of pGL4.10-4×E93^-623 to -606^ and pAc5.1/Flag-Kr-h1 was used as the control. Methoprene was applied at 10 μM. Means labeled with different letters indicate significant difference at *P*<0.05. *n*=4. **C** Upper panel: IP and WB showing interaction of Flag-Kr-h1, Flag-Kr-h1^S154A^ or Flag-Kr-h1^S154D^ with Flag-CBP. Mid and lower panels: the expression of above recombinant proteins in S2 cells. α-Kr-h1, Kr-h1 antibody; α-Flag, Flag antibody. WT, wildtype; MT, mutant. **D** Luciferase reporter assays after co-transfection of pGL4.10-4×RL36^-1647 to -1632^ and pAc5.1/Flag-CBP plus pAc5.1/Flag-Kr-h1, pAc5.1/Flag-Kr-h1^S154A^ or pAc5.1/Flag-Kr-h1^S154D^ into S2 cells. Co-transfection of pGL4.10-4×RL36^-1647 to -1632^ and pAc5.1/Flag-Kr-h1 was used as the control. Methoprene was applied at 10 μM. Means labeled with different letters indicate significant difference at *P*<0.05. *n*=4

Kr-h1 is known to act as a repressor and an activator in transcriptional response to JH [8, 11, 26, 27]. We performed ChIP analysis using the Kr-h1 antibody followed by LC-MS/MS as well as yeast two-hybrid assay to identify the co-factors of Kr-h1 in repressing nymphal metamorphosis and promoting adult reproduction. C-terminal binding protein (CtBP) is a highly conserved transcriptional corepressor involved in insect development and reproduction [54–56]. In *L. migratoria*, *CtBP* (GenBank: MT081314) expression was high in nymphs, but significantly decreased in adults (Additional file 1: Fig. S7A). Knockdown of *CtBP* caused significantly increased levels of *E93* transcript in penultimate 4th instar nymphs (Additional file 1: Fig. S7B), suggesting a crucial role of CtBP in repressing *E93* expression. To assess the p-Kr-h1 and CtBP interaction as well as the effect on *E93* transcription, Co-IP and luciferase reporter assays were performed using S2 cells co-transfected with recombinant pAc5.1/Flag-CtBP along with pAc5.1/Flag-Kr-h1, pAc5.1/Flag-Kr-h1^S154A^, or pAc5.1/Flag-Kr-h1^S154D^ plus pGL4.10-4×E93^-623 to -606^. Immunoprecipitation with anti-Kr-h1 antibody followed by western blot with anti-Flag antibody demonstrated that methoprene-exposed Flag-Kr-h1 and Flag-Kr-h1^S154D^ but not Flag-Kr-h1^S154A^ interacted with Flag-CtBP (Fig. 5A). Dual luciferase reporter assays showed that *E93* reporter activity was reduced by 47% and 50%, respectively when Flag-CtBP was co-expressed with Flag-Kr-h1 or Flag-Kr-h1^S154D^ (Fig. 5B). In contrast, co-expression of Flag-CtBP and Flag-Kr-h1^S154A^ had no significant inhibitory effect on *E93* reporter activity (Fig. 5B). The data suggest that phosphorylated Kr-h1 recruits a repressor, CtBP in transcriptional repression of *E93* gene for anti-metamorphic action in nymphal locusts.

CREB-binding protein (CBP), a transcriptional coactivator with histone acetyltransferase activity, has been demonstrated to play an important role in JH action [34, 57, 58]. In *L. migratoria*, *CBP* (GenBank: MT081315) mRNA levels significantly increased after adult ecdysis (Additional file 1: Fig. S7C). Depletion of *CBP* caused 49% reduction of *RL36* mRNA levels in the fat body of 3-day-old adult females (Additional file 1: Fig. S7D), suggesting that CBP is likely to participate in Kr-h1 regulation of *RL36* transcription. Co-IP assays showed that Flag-CBP dimerized with methoprene-treated Kr-h1 and Kr-h1^S154D^, but not Kr-h1^S154A^ (Fig. 5C). In dual luciferase reporter assays, co-transfection of pAc5.1/Flag-CBP and pAc5.1/Flag-Kr-h1^S154D^ caused 1.7-fold increase of *RL36* reporter activity, mimicking that observed with co-expression of Flag-CBP and Flag-Kr-h1 (Fig. 5D). Conversely, no significantly enhanced *RL36* reporter activity was observed with co-expression of Flag-CBP and Flag-Kr-h1^S154A^ (Fig. 5D). Taken together, these results imply that phosphorylated Kr-h1 recruits a coactivator, CBP for induction of *RL36* transcription that is involved in locust vitellogenesis and egg maturation.

### Kr-h1 phosphorylation is evolutionarily conserved

**Fig. 6:**
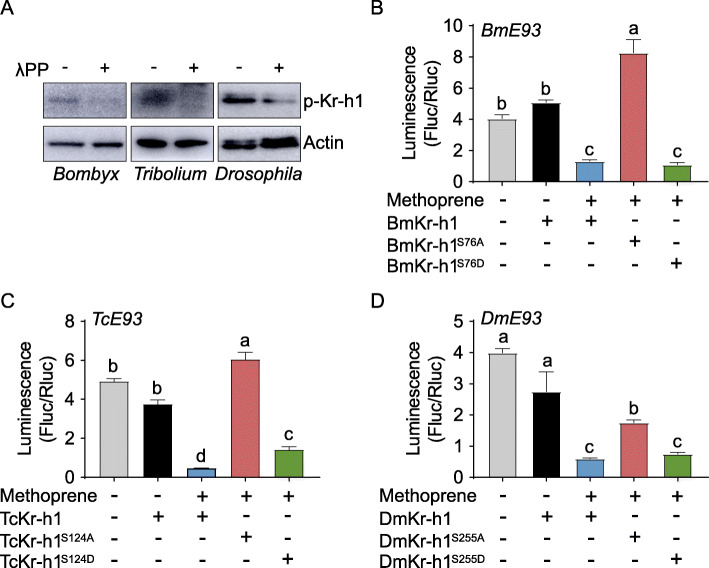
Conservation of Kr-h1 phosphorylation in other insects. **A** Western blot showing Kr-h1 phosphorylation in penultimate instar larvae of *Bombyx mori*, *Tribolium castaneum*, and *Drosophila melanogaster*, respectively. **B** Luciferase reporter assays after co-transfection of pGL4.10-4×BmE93^-2844 to -2827^ with pAc5.1/Flag-BmKr-h1, pAc5.1/Flag-BmKr-h1^S76A^, pAc5.1/Flag-BmKr-h1^S76D^ or pAc5.1/Flag vector control into S2 cells. Methoprene was applied at 10 μM. **C** Luciferase reporter assays using S2 cells co-transfected with pGL4.10-4×TcE93 ^-50 to -33^ with pAc5.1/Flag-TcKr-h1, pAc5.1/Flag-TcKr-h1^S124A^, pAc5.1/Flag-TcKr-h1^S124D^, or pAc5.1/Flag. **D** Luciferase reporter assays using S2 cells co-transfected with pGL4.10-4×DmE93^-2095 to -2078^ with pAc5.1/Flag-DmKr-h1, pAc5.1/Flag-DmKr-h1^S255A^, pAc5.1/Flag-DmKr-h1^S255D^ or pAc5.1/Flag. Means labeled with different letters indicate significant difference at *P*<0.05. *n*=3

We next investigated the evolutionary conservation of Kr-h1 phosphorylation across insect orders. Protein sequence alignment indicated that this phosphorylation residue is conserved in Kr-h1 orthologues of other 22 insect species with available cDNA sequences in the NCBI database (Additional file 1: Fig. S8A). We selected the Kr-h1 orthologues of holometabolous species *B. mori*, *T. castaneum*, and *D. melanogaster* for further study. Ser^154^ of *L. migratoria* Kr-h1 is homologous to Ser^76^ of *B. mori* Kr-h1 (BmKr-h1), Ser^124^ of *T. castaneum* Kr-h1 (TcKr-h1), and Ser^255^ of *D. melanogaster* Kr-h1 (DmKr-h1). Amino acids at the flanking regions of these serine residues occur in a highly conserved context (Additional file 1: Fig. S8A). The phosphorylated forms of Kr-h1 orthologues in *B. mori*, *T. castaneum* and *D. melanogaster* were recognized by anti-phospho-Kr-h1 (Ser^154^) antibody (Fig. 6A). The results indicate the conservation of Kr-h1 phosphorylation across insect orders, including hemimetabolous and holometabolous species. The regulatory sequences containing the core KBS motif were previously identified in the promoters of *B. mori*, *T. castaneum*, and *D. melanogaster E93* corresponding genes [17] (Additional file 1: Fig. S8B). Thus, we performed dual luciferase reporter assays to characterize the inhibitory effect of BmKr-h1, TcKr-h1, and DmKr-h1 phosphorylation on transcription of respective *E93* genes. Compared to the empty vector control, overexpression of methoprene-treated BmKr-h1 and BmKr-h1^S76D^ led to 67% and 73% reduction of *BmE93* reporter activity, whereas overexpression of BmKr-h1^S76A^ had no inhibitory effect (Fig. 6B). With respect to TcKr-h1 phosphorylation, methoprene-exposed TcKr-h1 and TcKr-h1^S124D^ caused 91% and 71% reduction, respectively of *TcE93* reporter activity (Fig. 6C). No inhibitory effect of TcKr-h1^S124A^ on *TcE93* reporter activity was observed (Fig. 6C). In the case of DmKr-h1 phosphorylation, methoprene-treated DmKr-h1 and DmKr-h1^S255D^ brought about 85% and 81% reduction, respectively, of *DmE93* reporter activity (Fig. 6D). Overexpression of DmKr-h1^S255A^ led to 44% reduction of *DmE93* reporter activity. Nevertheless, the transcriptional activity of DmKr-h1^S255A^ was significantly lower than that of methoprene-exposed DmKr-h1 and DmKr-h1^S255D^ (Fig. 6D). Collectively, these results indicate that Kr-h1 phosphorylation and its indispensable role in regulating *E93* expression are evolutionarily conserved in *B. mori*, *T. castaneum*, and *D. melanogaster*.

## Discussion

As a primary JH early-response gene, *Kr-h1* plays an essential role in mediating JH action in repressing metamorphosis in juveniles and stimulating reproduction in adults [8–11]. Previous studies have established that *Kr-h1* is transcriptionally activated by the JH-receptor complex [15, 29–31]. In addition, *Kr-h1* is reported to be post-transcriptionally regulated by miRNAs, including miR-2, let-7, and miR-278, in different species [37, 38]. Furthermore, *Kr-h1* transcription is regulated by HDAC1-mediated histone deacetylation, suggesting an epigenetic modification in JH action [34, 35]. Thus, Kr-h1 phosphorylation represents an interesting question for comprehensively deciphering the molecular basis of JH action and Kr-h1 function. By approaches of site-directed mutagenesis, phosphoprotein gel staining, LC-MS/MS, RNAi, western blot, and ChIP, we found in this study that Kr-h1 was phosphorylated by PKCα at Ser^154^ and that Kr-h1 phosphorylation levels increased along with JH-induced Kr-h1 expression. We observed more rapid increase of Kr-h1 phosphorylation than total Kr-h1 protein after 15-min exposure to methoprene in locusts. JH-induced phosphorylation was also seen with the recombinant Flag-Kr-h1 protein expressed in S2 cells. It has been previously reported that JH promotes Met phosphorylation by CaMKII and PKC and thus enhances the transcriptional activity of Met in *Ae. aegypti* [41, 43]. Moreover, JH triggers Akt-mediated serine/arginine-rich (pre-mRNA) splicing factor (SRSF) phosphorylation that induces Taiman alternative splicing and promotes *Ae. aegypti* vitellogenesis [40]. Additionally, it has been shown that JH induces Met phosphorylation and consequently increases the dimerization of Met and Tai in *H. armigera* [59]. Recently, a functional phosphorylation site (Ser^694^) located outside of multiple zinc-finger domains was identified in *Ae. aegypti* Kr-h1 (AaKr-h1). JH treatment caused dephosphorylation of AaKr-h1 at Ser^694^. Dephosphorylation mimic mutants (AaKr-h1^S694V^ and AaKr-h1^S694C^) showed significantly higher transcriptional activity than wildtype AaKr-h1 [60]. Our present study provides evidence on Kr-h1 phosphorylation at a serine residue in the zinc-finger domains and extends the view of post-translational modification of key players in the JH pathway. In a previous report, we demonstrated that JH activates the GPCR/RTK-PLC-IP3R signaling pathway that triggers PKC-mediated phosphorylation of Na^+^/K^+^-ATPase involved in patency induction and Vg transportation in vitellogenic female locusts [39]. We speculate that JH-activated GPCR/RTK-PLC-IP3R signaling cascade might induce PKCα-triggered Kr-h1 phosphorylation.

Kr-h1 is capable of activating or repressing transcription of genes in response to JH bound to its receptor Met [8, 11, 26, 27]. Our cell culture-based luciferase reporter assay and in vivo ChIP analysis demonstrated that Kr-h1 phosphorylation at Ser^154^ is essential for the transcriptional regulation of *E93* and *RL36*, two representatives of Kr-h1 target genes. Such a phosphorylation was required for Kr-h1 to interact with the corepressor CtBP in inhibiting *E93* transcription and with the coactivator CBP in inducing *RL36* transcription. The p-Kr-h1 wildtype variant Flag-Kr-h1^S154D^ had similar capability to p-Kr-h1 in binding cofactors and exerting transcriptional activity. However, the Kr-h1^S154A^ mutant was unable to recruit the cofactors, consequently abolishing the repression of *E93* transcription and the induction of *RL36* transcription. These results together address the importance of Kr-h1 phosphorylation in mediating anti-metamorphic and vitellogenic effects of JH.

The Kr-h1 sequence contains eight C_2_H_2_ zinc-finger domains. In addition to potentially recognizing a variety of DNA sequences, the zinc-fingers act as a hub for protein-protein interaction [61, 62]. The Ser^154^ residue is localized at the 3rd zinc-finger domain of Kr-h1. Phosphorylation modification is likely to induce a conformational change that is optimal for Kr-h1 to recruit cofactors. In the present study, CtBP and CBP were found to bind with phosphorylated Kr-h1 in repressing *E93* transcription and activating *RL36* transcription, respectively. Nevertheless, phosphorylated Kr-h1 could also interact with other cofactors in transcriptional activation or repression of target genes. In *Ae. aegypti*, Kr-h1 acts synergistically with Hairy, thereby mediating the action of Met in gene repression during previtellogenic development of adult females [27, 63]. A study in *N. lugens* has demonstrated that Hairy directly interacts with the N-terminus zinc-finger domains of Kr-h1 in modulating gene transcription [64]. Dual functions of transcriptional activation and repression are widely observed with transcription factors [65–70]. In mammals, Krüppel-like factor 4 promotes the transcription of *cyclin B1* via interacting with CBP, but downregulates *cyclin B1* transcription by recruiting HDAC3 [66].

We have additionally shown Kr-h1 phosphorylation in other insects belonging to divergent orders, including the lepidopteran *B. mori*, the coleopteran *T. castaneum*, and the dipteran *D. melanogaster*. The requirement of phosphorylation for Kr-h1 action on suppressing *E93* transcription was found to be also conserved. The findings provide a clear indication that Kr-h1 phosphorylation and its indispensable role in regulating target gene expression are evolutionarily conserved across distant insect orders. These observations further highlight the significance of Kr-h1 phosphorylation in eliciting transcriptional activity. Previously, JH-dependent *Ae. aegypti* Kr-h1 dephosphorylation at Ser^694^ has been demonstrated to enhance the transcriptional activity [60]. The phosphoserine residue Ser^694^ is conserved in some holometabolous insects but not in *L. migratoria*. The Ser^154^ of *L. migratoria* Kr-h1 is homologous to Ser^206^ of *Ae. aegypti* Kr-h1. Thus, Kr-h1 orthologues likely bear multiple phosphorylation sites with differential responses to JH. While evolutionarily conserved Kr-h1 phosphorylation sites occur in divergent insect species, the lineage- and species-specific Kr-h1 phosphorylation residues may exist in some insects. It is of interest to address these questions in future research.

## Conclusions

Kr-h1 functions both as a transcriptional repressor in preventing precocious larval/nymphal metamorphosis and a transcriptional activator in stimulating adult reproduction in insects. PKCα phosphorylated Kr-h1 at a serine residue localized in the 3^rd^ zinc-finger domain. While Kr-h1 phosphorylation levels increased along with JH-induced total Kr-h1 expression, more rapid increase of Kr-h1 phosphorylation than total Kr-h1 was observed in locusts treated with methoprene. JH-induced Kr-h1 phosphorylation was also seen in methoprene-exposed S2 cells. Phosphorylated Kr-h1 recruited CtBP in nymphs, which inhibited *E93* expression and metamorphosis. Phosphorylated Kr-h1 recruited CBP in adults, consequently stimulating *RL36* transcription and vitellogenesis. Kr-h1 phosphorylation and its essential role in recruiting CtBP and repressing *E93* expression are evolutionarily conserved in *L. migratoria*, *B. mori*, *T. castaneum*, and *D. melanogaster*. Thus, our present study fills a knowledge gap of phosphorylation modification of Kr-h1, an intermediate regulator in the JH/Met-response gene expression hierarchy.

## Methods

### Insects and treatments

The gregarious phase of *L. migratoria* was maintained as previously reported [71]. s-(+)-methoprene (Santa Cruz Biotech) was topically applied at 10–100 μg/5 μl acetone per locust for 8 h or 100 μg/5μl acetone per locust for 5-60 min. NPC15437 (Abcam) was intra-abdominally injected at 0.25 μg/5 μl DMSO per locust.

### LC-MS/MS analysis

Synthesized Kr-h1(aa125-159) and Kr-h1^S154A^(aa125-159) peptides (BiotechPark) were separately incubated with PKCα (SignalChem) in reaction buffer containing 50 mM Tris-HCl (pH 7.5), 100 mM NaCl, 20 mM MgCl_2_, 1 mM DTT, and 1 mM ATP at 30°C for 30 min. After termination with 1/10 volume 1% formaldehyde and centrifugation at 8000×*g* for 10 min, the supernatant was desalted by C18Zip-Tip (Millipore), reduced by 10 mM DTT at 56°C for 1 h, and alkylated by 20 mM iodoacetamide (IAA) at room temperature in dark for 1 h. Extracted peptides were then lyophilized and resuspended in 0.1% formic acid, followed by LC-MS/MS analysis.

### Pro-Q Diamond Phosphoprotein Gel Stain

cDNA fragments for Kr-h1(aa1-290), Kr-h1^S154A^(aa1-290), Kr-h1(aa89-312), Kr-h1^S154A^(aa89-312), and Kr-h1(aa291-591) were separately cloned into pGEX-4t-1 vector (GE Healthcare) for overexpression of recombinant GST-tagged proteins in *Escherichia coli* Rosetta competent cells (Transgen). Cells were lysed by sonication in lysis buffer with 50 mM Tris-HCl pH 7.5 plus 0.1% Triton X-100 and cleared by centrifugation at 8000×*g* for 30 min at 4°C. GST-fusion proteins were purified by GST resin (Thermo Fisher Scientific) and incubated with PKCα (SignalChem), followed by SDS-PAGE and Pro-Q Diamond Phosphoprotein Gel Stain (Invitrogen).

### Eukaryotic cell culture and protein expression

Protein coding sequences of Kr-h1 (nt 1-1776), CtBP (nt 1-1332), CBP (nt 1-1728), BmKr-h1 (nt 1-1086), TcKr-h1 (nt 1-1407), and DmKr-h1 (nt 1-2376) were separately cloned into pAc5.1/Flag vectors (Invitrogen). Site-directed mutagenesis for Kr-h1^S154A^, Kr-h1^S154D^, BmKr-h1^S76A^, BmKr-h1^S76D^, TcKr-h1^S124A^, TcKr-h1^S124D^, DmKr-h1^S255A^, and DmKr-h1^S255D^ was performed using Q5 Site-Directed Mutagenesis Kit (NEB). S2 cells were transfected with the recombinant vectors using Lipofectamine 3000 (Thermo). Primers used for recombinant vector construction and site-directed mutagenesis are provided in Table S1 (Additional file 1) and Table S2 (Additional file 1), respectively.

### Western blot and immunoprecipitation

Protein extracts from insects and S2 cells were isolated in ice-cold lysis buffer containing 150 mM NaCl, 50 mM Tris-HCl (pH 7.4), 1 mM EDTA, 1% Nonidet P-40, 1% Triton-X 100, 0.5% sodium deoxycholate, 1 mM PMSF plus protease, and phosphatase inhibitors (Roche). Lysates were cleared by centrifugation, subjected to 8% SDS-PAGE, and transferred to PVDF membrane (Millipore). Western blots were conducted using antibodies against Kr-h1 [38], phospho-Kr-h1 (Ser^154^) (Jingjie PTM-Biolab), VgA [39] and Flag (MBL), corresponding HRP-conjugated secondary antibody (CWBIO), and a Superstar ECL Plus Ready-to-use Kit (BOSTER). β-actin antibody [39] was used as a reference control. Band intensity was quantified by ImageJ. For immunoprecipitation, precleared lysates were incubated with anti-Kr-h1 antibody for 60 min at 4°C. The immunocomplexes were then captured with protein-A agarose (Sigma-Aldrich) at 4°C overnight and eluted in Laemmli sample buffer, followed by western blots with anti-phospho-(Ser) (Blue Light Biotech) or anti-Flag antibody. For phosphatase treatment, protein extracts were preincubated with λpp (New England Biolabs) for 1 h at 30°C.

### RNA isolation and qRT-PCR

Total RNAs were extracted from insects and S2 cells using TRIzol reagent (Invitrogen), and first-strand cDNAs were reverse transcribed using FastQuant RT kit with gDNase (Tiangen). qRT-PCR was performed using a RealMasterMix SYBR Green kit (Tiangen) with a LightCycler 96 system (Roche), initiated at 95°C for 15 min, and followed by 40 cycles of 95°C for 10 s, 58°C for 20 s, and 72°C for 30 s. Relative expression levels were calculated using 2^−ΔΔCt^ method, normalized by ribosomal protein 49 (Rp49). Primers for qRT-PCR are listed in Table S3 (Additional file 1).

### RNA interference and tissue imaging

cDNA templates were amplified by PCR, cloned into pGEM-T vector (Tiangen), and confirmed by sequencing. dsRNAs were synthesized by in vitro transcription with T7 RiboMAX Express RNAi System (Promega). Locusts were intra-abdominally injected with 15 μg dsRNA, and boosted once on day 5. Phenotypes were photographed by Canon EOS550D camera and Leica M205C stereomicroscope. Primers used for RNAi are given in Table S3 (Additional file 1).

### Dual luciferase reporter assay

*E93* and *RL36* promoter regions bearing the KBS motif including 4×E93^-623 to -606^, 4×RL36^-1647 to -1632^, 4×BmE93^-2844 to -2827^, 4×TcE93^-50 to -33^, and 4×DmE93^-2095 to -2078^ were separately ligated into pGL4.10 vector (Promega) and confirmed by sequencing. S2 cells were co-transfected with these constructs along with recombinant vectors expressing wildtype or mutated Kr-h1 of *L. migratoria*, *B. mori*, *T. castaneum*, and *D. melanogaster*. Methoprene was applied at 10 μM 48 h post transfection and for 6 h. The luciferase activity was measured using a Dual-Luciferase Reporter Assay System and a GloMAX 96 Microplate Luminometer (Promega).

### Chromatin immunoprecipitation

ChIP assays were performed using an EZ-Magna ChIP A/G Kit (Millipore). Briefly, fat bodies collected from nymph and adult females were fixed with 1% formaldehyde to crosslink chromatin for 10 min at 37°C. After addition of 125 mM glycine, chromatin was sonicated to shear into 200-1000 bp DNA fragments. The complexes were then immunoprecipitated with antibody against Kr-h1, phospho-Kr-h1 (Ser^154^) or IgG, followed by qPCR. Primers used for ChIP are listed in Table S3 (Additional file 1).

### Statistical analysis

Statistical analyses were performed by Student’s *t*-test or one-way analysis of variance (ANOVA) with Tukey’s post hoc test using the SPSS22.0 software. Significant difference was considered at *P* < 0.05. Values were reported as mean ± S.E.

## Supplementary Information

**Additional file 1: Figure S1.** Identification of Kr-h1 phosphorylation site. **Figure S2.** Identification of kinase triggering Kr-h1 phosphorylation. **Figure S3.** Effect of 15-min exposure of methoprene on Kr-h1 phosphorylation. **Figure S4.** Effect of *E93* knockdown on locust metamorphosis. **Figure S5.** Responsiveness of Kr-h1 phosphorylation to JH. **Figure S6.** Effect of *RL36* knockdown on locust reproduction. **Figure S7.** Effect of *CtBP* or *CBP* knockdown on *E93* or *RL36* expression. **Figure S8.** Alignment of the 3^rd^ zinc-finger domain of Kr-h1 and the partial promoter sequences of *E93* with KBS motifs. **Table S1.** Primers used for cloning and gene expression. **Table S2.** Primers used for site-directed mutagenesis. **Table S3.** Primers used for qRT-PCR, RNAi and ChIP.

**Additional file 2.** The individual data values for Fig. 3B-E, Fig. 4B-E, Fig. 5B, D, Fig. 6B-D, Fig. S2A and Fig. S3.

**Additional file 3.** Original Western blot data

## Data Availability

All data generated or analyzed during this study are included in this published article and its supplementary information files. The datasets used and/or analyzed during the current study available from the corresponding author on reasonable request.
