# Supplementary material for: Krüppel-homolog 1 exerts anti-metamorphic and vitellogenic functions in insects via phosphorylation-mediated recruitment of specific cofactors
Source: BMC Biol. 2021 Oct 8;19:222. doi: 10.1186/s12915-021-01157-3 (PMC8499471; doi:10.1186/s12915-021-01157-3)
Supplement: Supplementary file 1 — Additional file 1: Figure S1. Identification of Kr-h1 phosphorylation site. Figure S2. Identification of kinase triggering Kr-h1 phosphorylation. Figure S3. Effect of 15-min exposure of methoprene on Kr-h1 phosphorylation. Figure S4. Effect of E93 knockdown on locust metamorphosis. Figure S5. Responsiveness of Kr-h1 phosphorylation to JH. Figure S6. Effect of RL36 knockdown on locust reproduction. Figure S7. Effect of CtBP or CBP knockdown on E93 or RL36 expression. Figure S8. Alignment of the 3rd zinc-finger domain of Kr-h1 and the partial promoter sequences of E93 with KBS motifs. Table S1. Primers used for cloning and gene expression. Table S2. Primers used for site-directed mutagenesis. Table S3. Primers used for qRT-PCR, RNAi and ChIP. [file 12915_2021_1157_MOESM1_ESM.pdf]

Additional file 1：

Krüppel-homolog 1 exerts anti-metamorphic and vitellogenic functions in insects via phosphorylation-mediated recruitment of specific cofactors

Zhongxia Wu, Libin Yang, Huihui Li, Shutang Zhou*

State Key Laboratory of Cotton Biology, Key Laboratory of Plant Stress Biology, School of Life Sciences, Henan University, Kaifeng 475004, China

*Corresponding author:

Shutang Zhou, PhD, Professor

State Key Laboratory of Cotton Biology, Key Laboratory of Plant Stress Biology, School of Life Sciences, Henan University, Kaifeng 475004, China

Tel: +86-371-23886272

Email: szhou@henu.edu.cn

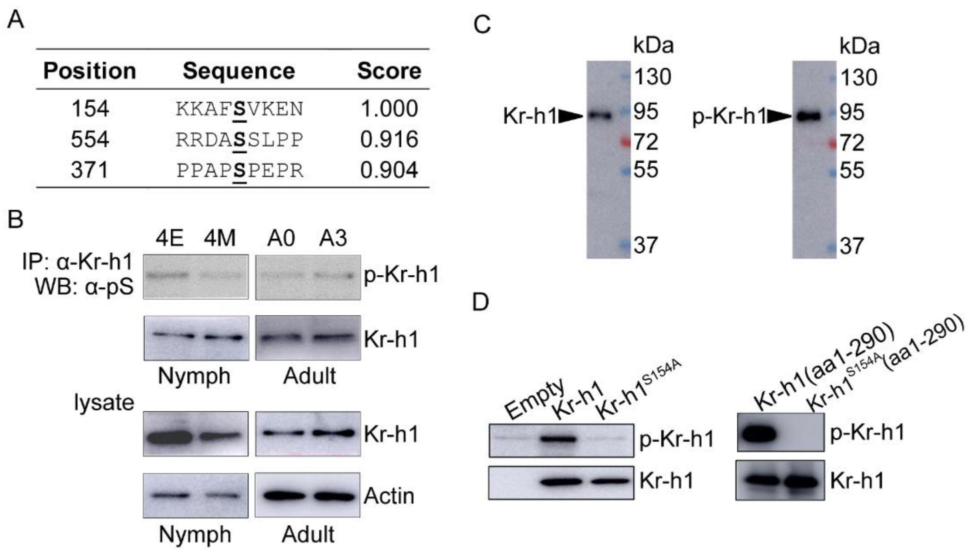

Additional file 1: Figure S1. Identification of Kr-h1 phosphorylation site. (A) Top three Kr-h1 phosphorylation sites predicted by the DISPHOS (V1.3) software. (B) Immunoprecipitation (IP) and western blot (WB) showing the abundance of Kr-h1 and phosphorylated Kr-h1 (p-Kr-h1) in the whole body of early (4E) and mid (4M) penultimate 4th instar nymphs as well as the fat body of adult female locusts on day 0 (A0) and 3 (A3). α-pS, phospho-(Ser) antibody; α-Kr-h1, Kr-h1 antibody. (C) Western blots with both anti-Kr-h1and anti-p-Kr-h1 antibodies (arrows) shown in full extent with molecular weight markers (color bands). (D) Verification of phospho-Kr-h1 (Ser^{154}) antibody specificity by western blots. Left panel: Flag-Kr-h1 and mutated Kr-h1^{S154A} expressed in *Drosophila* S2 cells treated with 10 μM methoprene. Right panel: the bacterially-expressed GST-tagged peptides of Kr-h1(aa1-290) and Kr-h1^{S154A}(aa1-290) preincubated with PKCα.

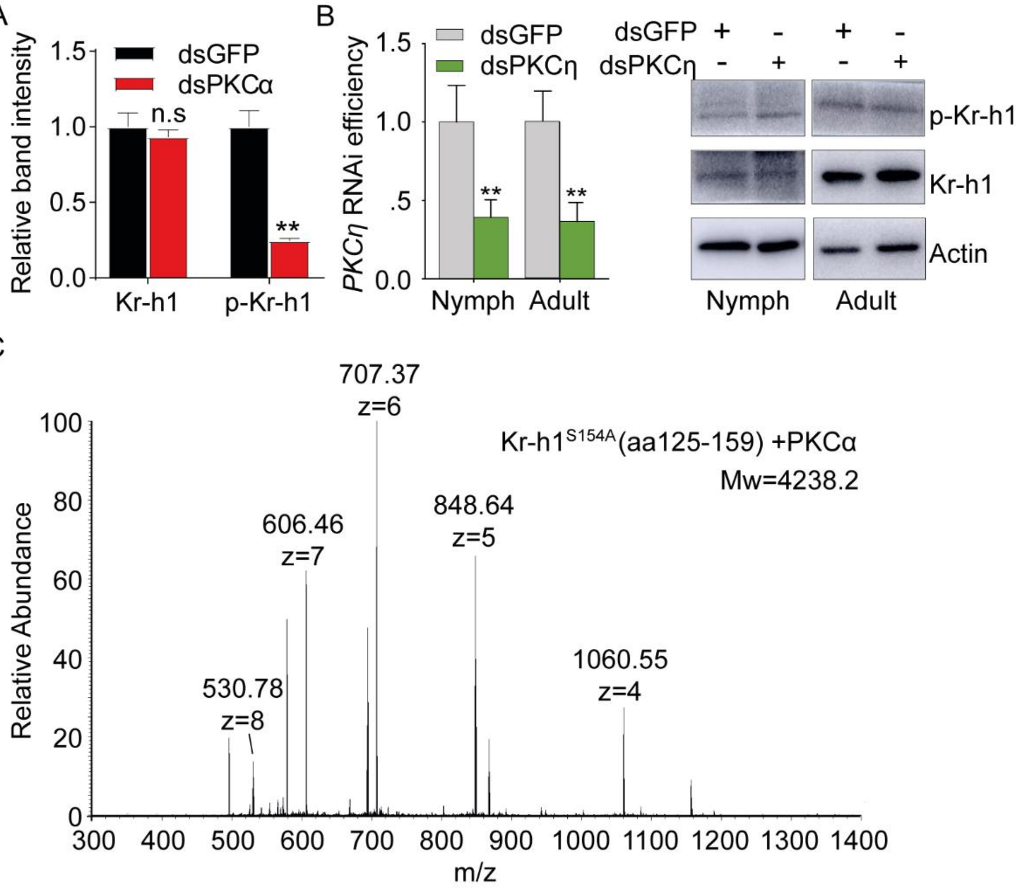

Additional file 1: Figure S2. Identification of kinase triggering Kr-h1 phosphorylation. (A) Quantitative analysis of Kr-h1 and p-Kr-h1 band intensity in western blots using protein extracts from the fat body of adult females injected with dsPKCα vs. dsGFP controls (represented by Figure 1B right panel). **, P<0.01. n=4. (B) Effect of *PKCη* knockdown on Kr-h1 phosphorylation. Left panel: *PKCη* knockdown efficiency. Right panels: the levels of Kr-h1 and p-Kr-h1. Nymph, the whole body of mid penultimate 4th instar nymphs. Adult, the fat body of 3-day-old adult females. **, P<0.01. n=8. (C) LC-MS/MS analysis of mutated Kr-h1^{S154A}(aa125-159) peptide preincubated with PKCα. m/z indicates the mass to charge ratio.

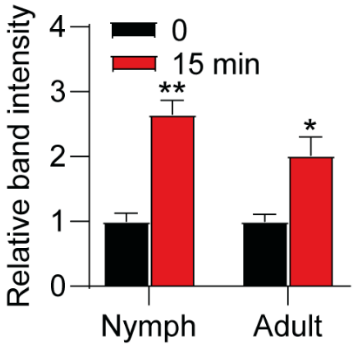

Additional file 1: Figure S3. Effect of 15-min exposure of methoprene on Kr-h1 phosphorylation. Quantitative analysis of p-Kr-h1 band intensity in western blots using the whole body of mid 5th instar nymphs and the fat body from newly-emerged adult females treated with 100 μg methoprene for 15 min (represented by Figure 2B and 2E, respectively). *, $P<0.01$; **, $P<0.01$; n=3.

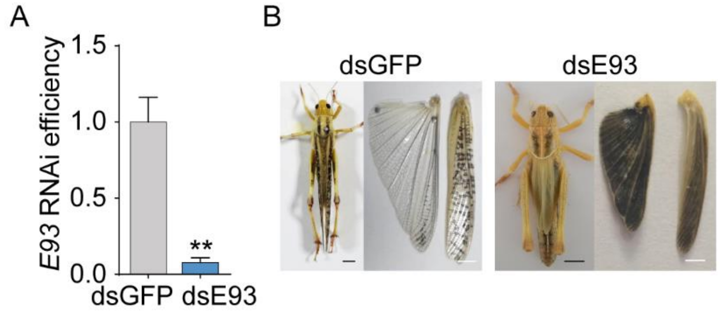

Additional file 1: Figure S4. Effect of *E93* knockdown on locust metamorphosis. (A) *E93* RNAi efficiency in the final instar nymphs. **, *P*<0.01. n=8. (B) Representative phenotypes of delayed adult eclosion (super nymphs) after *E93* knockdown. Scale bar: black, 5 mm; white, 0.5 mm.

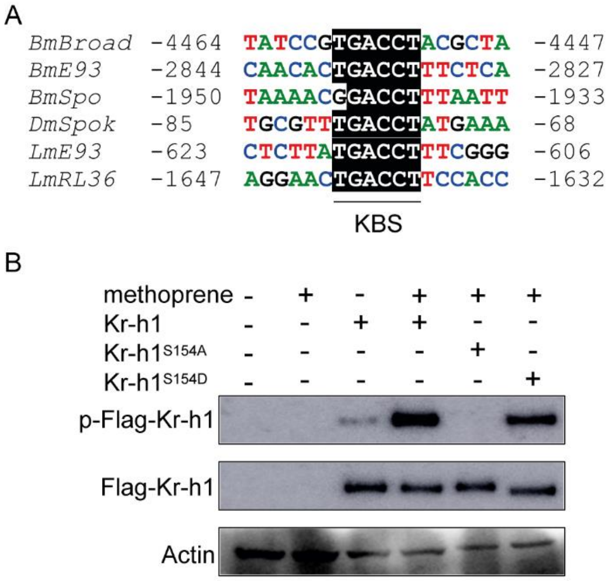

Additional file 1: Figure S5. Responsiveness of Kr-h1 phosphorylation to JH. (A) Alignment of DNA sequences containing the core KBS motif in the upstream of *Br-C*, *E93* and *Spo* from the silkworm *Bombyx mori* (*Bm*), *Spok* from *Drosophila melanogaster* (*Dm*) (references 13, 17 and 18), as well as *E93* (GenBank: MT081312) and *RL36* (GenBank: MT081313) from *Locusta migratoria* (*Lm*). (B) Western blot showing the expression of recombinant Flag-Kr-h1, Flag-Kr-h1^{S154A} and Flag-Kr-h1^{S154D} in *Drosophila* S2 cells with or without 10 µM methoprene treatment. p-Kr-h1, phospho-Kr-h1 (Ser^{154}) antibody. Flag-Kr-h1, Kr-h1 antibody.

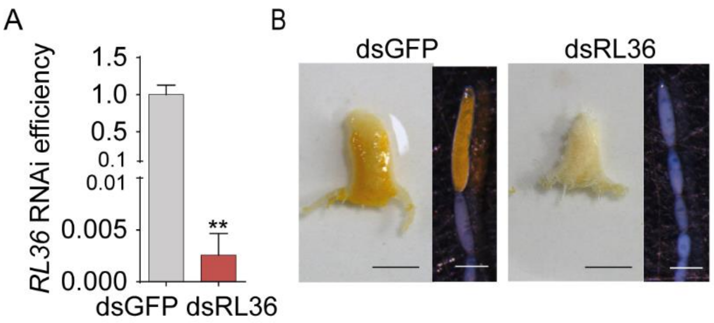

Additional file 1: Figure S6. Effect of *RL36* knockdown on locust reproduction. (A) *RL36* RNAi efficiency in the fat body of 6-day-old adult females. **, $P<0.01$. n=8. (B) Representative phenotypes of ovaries and primary oocytes after *RL36* knockdown. Scale bar: black, 1 cm; white, 1 mm.

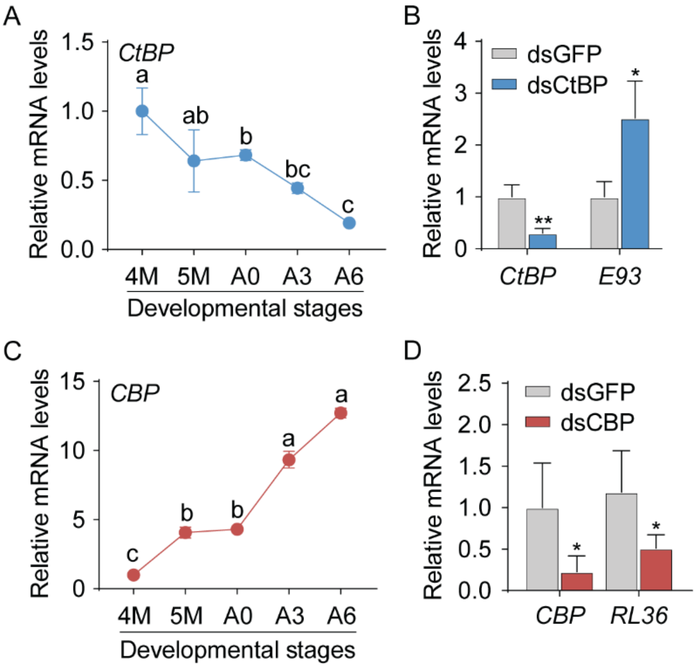

Additional file 1: Figure S7. Effect of *CtBP* or *CBP* knockdown on *E93* or *RL36* expression. (A) Developmental profiles of *CtBP* in the whole body of mid 4th (4M) and 5th (5M) instar nymphs as well as adult females on day 0 (A0), 3 (A3) and 6 (A6). Means labeled with different letters indicate significant difference at $P<0.05$. n=8. (B) Effect of *CtBP* knockdown on the expression of *E93* in mid 4th instar nymphs. *, $P<0.05$ and **, $P<0.01$. n=8. (C) Developmental dynamics of *CBP* in the fat body of 4M and 5M instar nymphs as well as A0, A3 and A6 adult females. Means labeled with different letters indicate significant difference at $P<0.05$. n=8. (D) Effect of *CBP* knockdown on the expression of *RL36* in the fat body of 3-day-old adult females. *, $P<0.05$. n=8.

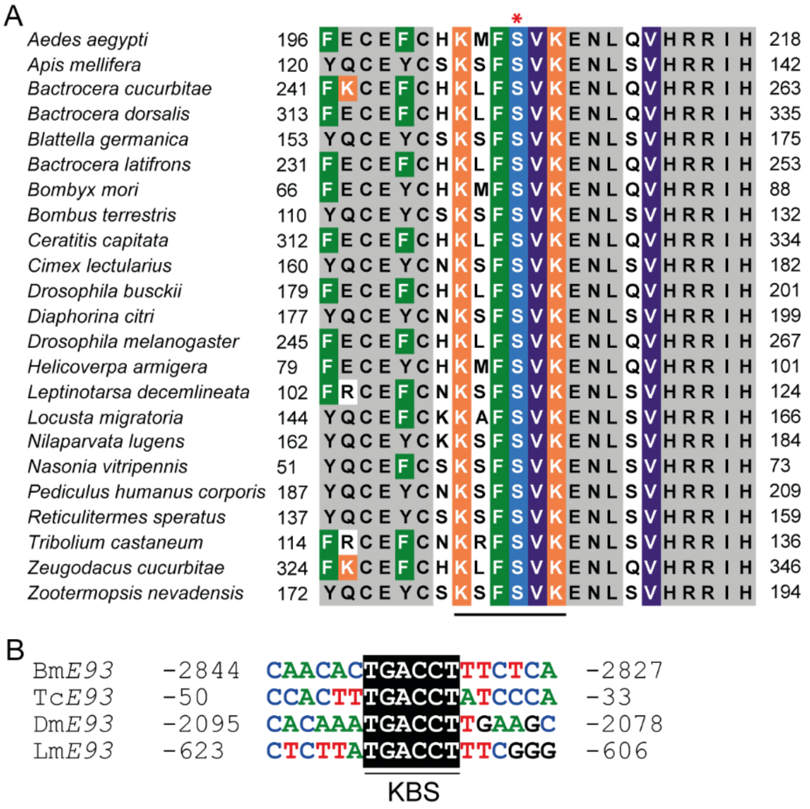

Additional file 1: Figure S8. Alignment of the 3rd zinc-finger domain of Kr-h1 and the partial promoter sequences of *E93* with KBS motifs. (A) Alignment of the 3rd zinc-finger domain of Kr-h1 from 23 insect species with available cDNA sequences in NCBI database. Numbers donate starting and ending positions. Asterisk indicates the conserved serine residues homology to Ser154 of locust Kr-h1. Underlined amino acids indicate the conserved motif recognized by PKC. (B) Alignment of DNA sequences containing the core KBS motif in the promoters of *E93* from *Bombyx mori* (Bm), *Tribolium castaneum* (Tc) (GenBank: XM_015983777), *Drosophila melanogaster* (Dm) (FlyBase: FBgn0264490) and *Locusta migratoria* (Lm) (GenBank: MT081312).

Additional file 1: Table S1. Primers used for cloning and gene expression

**Gene**	**Forward primer (5' to 3')**	**Reverse primer (5' to 3')**
Kr-h1	ATGGTGGGCTACTTCAACGG	TTACGAGGCGCCCGCGTAGT
Kr-h1(aa1-290)	ATGGTGGGCTACTTCAACGG	GCTGCAGATGGTGCACTTGT
Kr-h1(aa89-312)	TCCTTCTGCCAGAAGACGTT	GGGGTGCTGCTGCTCCGAGT
Kr-h1(aa291-591)	GAGACGTTCGCCTCCAAGAA	TTACGAGGCGCCCGCGTAGT
CtBP	ATGGACAAGCGCAAGATGCT	TTAATGTACTTCTGATGGCTC
CBP	GATGGAGTGGATGTATGCTT	TTAACCAGGAGGATTTCCTTTC
BmKr-h1	ATGGAATCATTACCTATTTT	CTATGATTCTGTAGCTGGCG
DmKr-h1	ATGGTTTACTATTCCGCCA	CTAGGAGGCCTTGGCGAA
TcKr-h1	ATGCCGGAAATGGTCGGT	ACGACGCTCCTGCATATTG

Additional file 1: Table S2. Primers used for site-directed mutagenesis

Mutation	Primer sequence (5’ to 3’)
Kr-h1^{S154A}-F	AAGGCCTTCGCCGTCAAGGAGAACCTGAGCGTGCACCGGCG
Kr-h1^{S154A}-R	TCTCCTTGACGGCGAAGGCCTTCTTGCAGAACTCGCACTGG
Kr-h1^{S371A}-F	GCCGGCGCCGGCGCCGGAGCCCCGCCTCGAACCCCGCCTCG
Kr-h1^{S371A}-R	GGGCTCCGGCGCCGGCGCCGGCGGCGGAGGTAGGTGCTGTT
Kr-h1^{S554A}-F	CGCGACGCCGCCTCGCTGCCGCCCCGCAAGCGCTGCAAGG
Kr-h1^{S554A}-R	GGCAGCGAGGCGGCGTCGCGGCGGTCGGGCGACGCGGGGG
Kr-h1^{S154D}-F	AAGGCCTTCGACGTCAAGGAGAACCTGAGCGTGCACCGGC
Kr-h1^{S154D}-R	TCTCCTTGACGTCGAAGGCCTTCTTGCAGAACTCGCACTGGT
BmKr-h1^{S76A}-F	AAATGTTTGCTGTGAAAGAAAATTTGCAAGTACACCGTC
BmKr-h1^{S76A}-R	TCTTTCACAGCAAACATTTTATGGCAATATTCACATTCA
BmKr-h1^{S76D}-F	AAATGTTTGATGTGAAAGAAAATTTGCAAGTACACCGTC
BmKr-h1^{S76D}-R	TCTTTCACATCAAACATTTTATGGCAATATTCACATTCA
DmKr-h1^{S255A}-F	CACAAGCTGTTCGCCGTGAAGGAGAACCTCCAGGTGCAC
DmKr-h1^{S255A}-R	CTTCACGGCGAACAGCTTGTGGCAGAACTCGCACTCGAA
DmKr-h1^{S255D}-F	CACAAGCTGTTCGACGTGAAGGAGAACCTCCAGGTGCAC
DmKr-h1^{S255D}-R	CTTCACGTCGAACAGCTTGTGGCAGAACTCGCACTCGAA
TcKr-h1^{S124A}-F	AGCGATTCGCCGTTAAAGAAAACTTGAGCGTTCATCGAAGA
TcKr-h1^{S124A}-R	TCTTTAACGGCGAATCGCTTATTGCAAAATTCGCAACGGAA
TcKr-h1^{S124D}-F	AGCGATTCGACGTTAAAGAAAACTTGAGCGTTCATCGAAGA
TcKr-h1^{S124D}-R	TCTTTAACGTCGAATCGCTTATTGCAAAATTCGCAACGGAA

Additional file 1: Table S3. Primers used for qRT-PCR, RNAi and ChIP

	Gene	Forward primer (5' to 3')	Reverse primer (5' to 3')
**qRT-PCR**	*Kr-h1*	AGTGCCAGGTGTGCTCCAAGA	CGAACGACTTGCCGCAGATGT
	E93	CAGGCTGGCGATGACAACA	AGTCCGATGGCGTGCTACT
	RL36	ACGAATGTGTGTGCCAAGC	CGTCCGTCAAGACTAAAGGG
	PKCα	AAGGCTCGGTTGTGGAACA	AGGAGGTTGGACTTCACGAT
	PKCη	AGCAACCAGCAACAAGAGGA	TCGTTCTGCCAGCATCACTT
	CtBP	GCGGTTGGTGCCCTAATGTG	TGAGGCAAAGGGTGGTGTCT
	CBP	TGCGTGTCAATGCCGTGATG	CTGCTGAACCTGCTCCACCA
	Rp49	CGTAAACCGAAGGGAATTGA	GAAGAAACTGCATGGGCAAT
**RNAi**	*Kr-h1*	GTCAAGGAGAACCTGAGCGTGC	TGCTGCTGCTCCGAGTGGCT
	PKCα	CGTTCCTCCCTTAACCCTGT	AACCTTTCCAAAGCTGCCTT
	PKCη	GCAGGCGTGTCCATCAAGTA	GGAGGTGTGAGCTTGTCTGT
	E93	AGGCTGGCGATGACAACACT	GCTGCACGGCGAGTTCCTAA
	RL36	GAAGGGACACCGGACGACAA	GTGTGTGCCAAGCCTCCTCT
	CtBP	TGGTGAACACAGCACGAGGT	ACGGAGTGTGGCACTTGGTC
	CBP	TGCGTGTCAATGCCGTGATG	CTGCTGAACCTGCTCCACCA
	GFP	CACAAGTTCAGCGTGTCCG	GTTCACCTTGATGCCGTTC
**ChIP**	*E93*	CCGTGGCAAGCTCGTTTT	GCACTTGGGGCAAACTGT
	RL36	GGAGAGTCAGTAGTAACTG	CAGCAGCCAAACTCTCTT
