# Supplementary material for: Krüppel-homolog 1 exerts anti-metamorphic and vitellogenic functions in insects via phosphorylation-mediated recruitment of specific cofactors
Source: BMC Biol. 2021 Oct 8;19:222. doi: 10.1186/s12915-021-01157-3 (PMC8499471; doi:10.1186/s12915-021-01157-3)
Supplement: Supplementary file 3 — Additional file 3. Original Western blot data [file 12915_2021_1157_MOESM3_ESM.pdf]

Figure 1

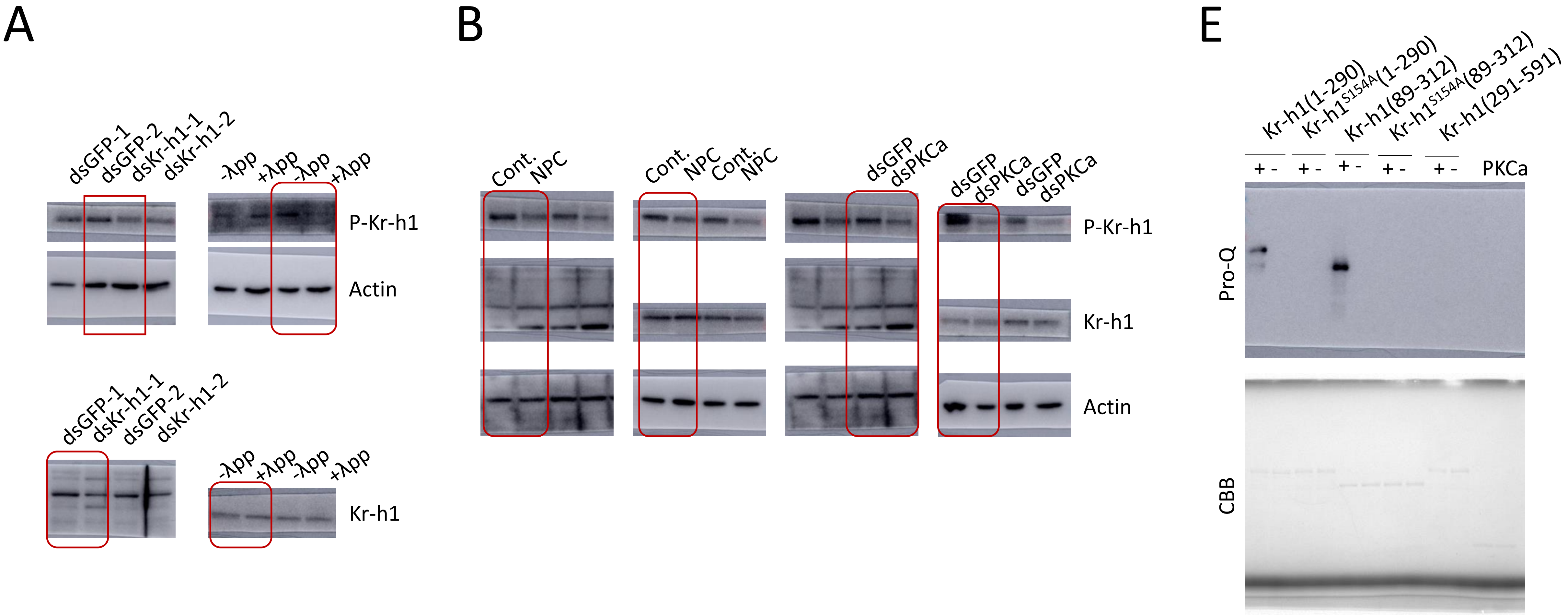
A
dsGFP-1
dsGFP-2
dsKr-h1-1
dsKr-h1-2
-λpp
+λpp
-λpp
+λpp
P-Kr-h1
Actin
dsGFP-1
dsKr-h1-1
dsGFP-2
dsKr-h1-2
-λpp
+λpp
-λpp
+λpp
Kr-h1
B
Cont.
NPC
Cont.
NPC
Cont.
NPC
dsGFP
dsPKCa
dsGFP
dsPKCa
dsGFP
dsPKCa
P-Kr-h1
Kr-h1
Actin
E
Kr-h1(1-290)
Kr-h1S154A(1-290)
Kr-h1(89-312)
Kr-h1S154A(89-312)
Kr-h1(291-591)
+ -
+ -
+ -
+ -
+ -
PKCa
Pro-Q
CBB

Figure 2

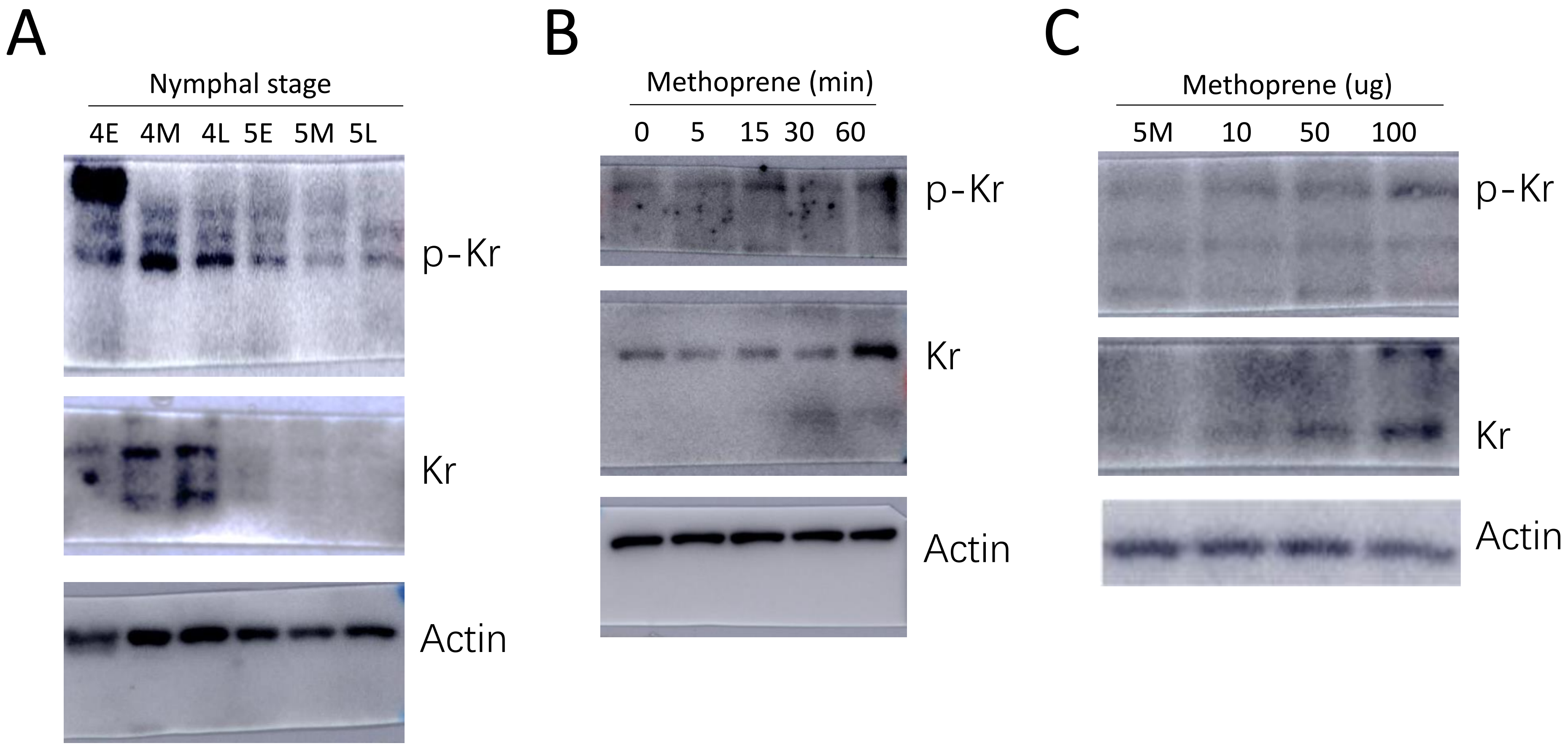
A
Nymphal stage
4E 4M 4L 5E 5M 5L
p-Kr
Kr
Actin
B
Methoprene (min)
0 5 15 30 60
p-Kr
Kr
Actin
C
Methoprene (ug)
5M 10 50 100
p-Kr
Kr
Actin

Figure 2

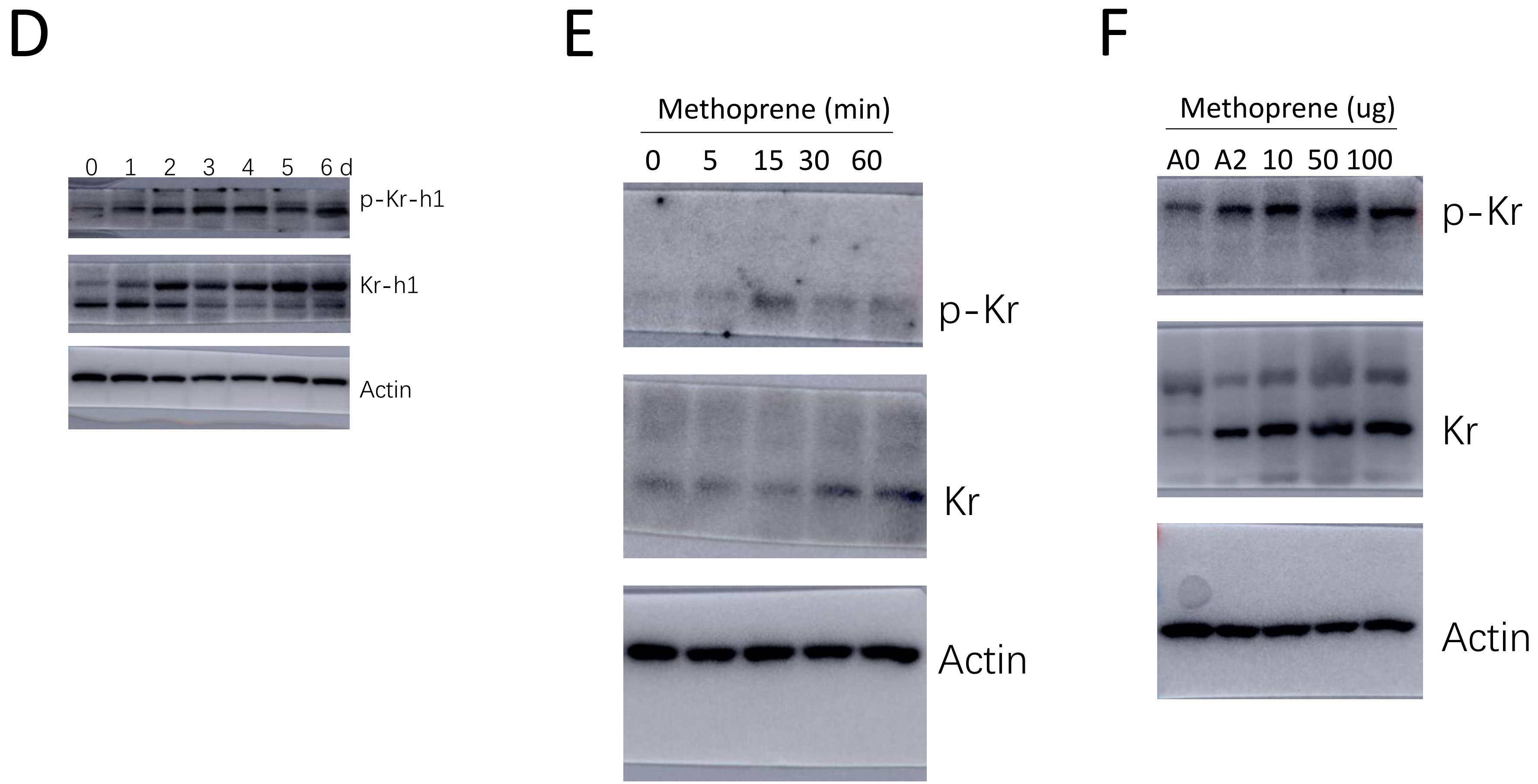

D
0 1 2 3 4 5 6 d
p-Kr-h1
Kr-h1
Actin
E
Methoprene (min)
0 5 15 30 60
p-Kr
Kr
Actin
F
Methoprene (ug)
A0 A2 10 50 100
p-Kr
Kr
Actin

Figure 5

A

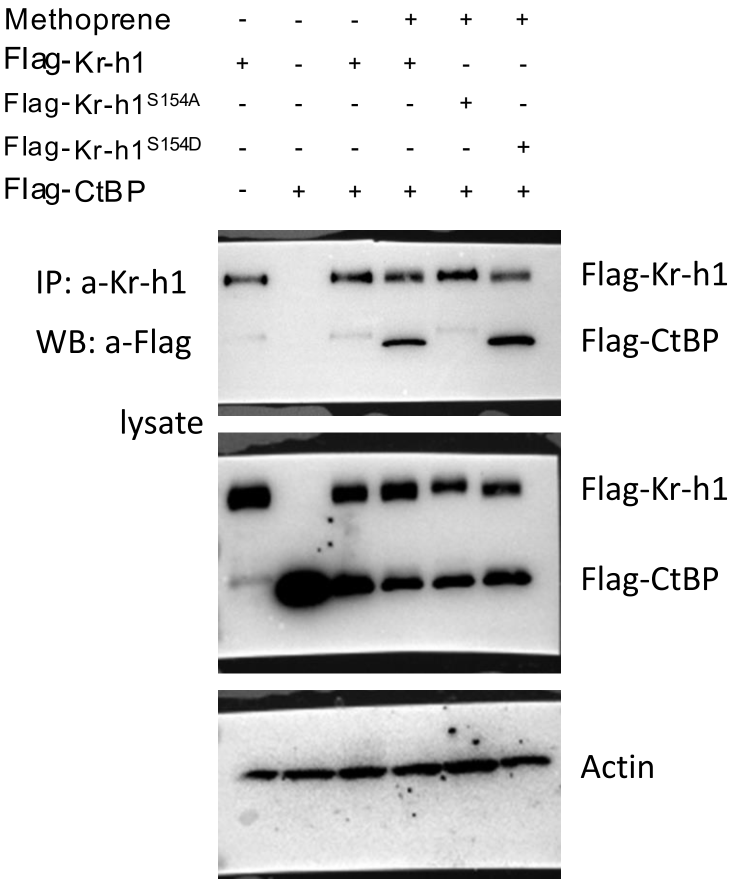

Methoprene - - - + + +
Flag-Kr-h1 + - + + - -
Flag-Kr-h1S154A - - - - + -
Flag-Kr-h1S154D - - - - - +
Flag-CtBP - + + + + +
IP: a-Kr-h1
WB: a-Flag
Flag-Kr-h1
Flag-CtBP
lysate
Flag-Kr-h1
Flag-CtBP
Actin

C

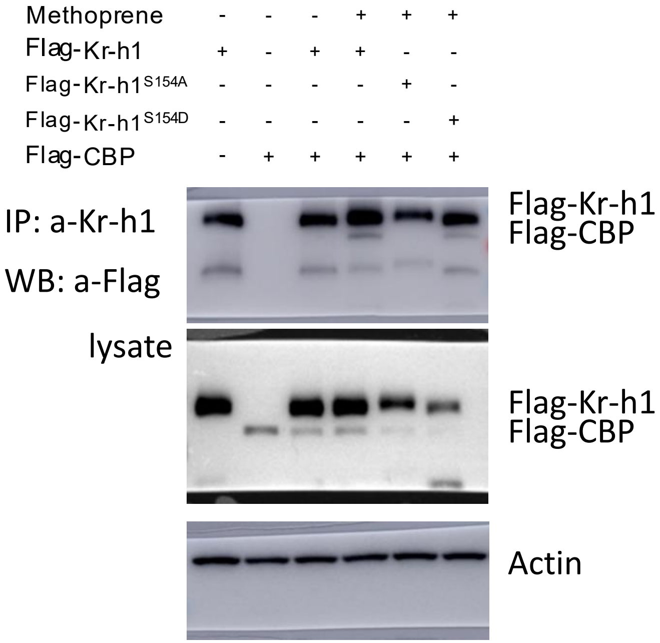

Methoprene - - - + + +
Flag-Kr-h1 + - + + - -
Flag-Kr-h1S154A - - - - + -
Flag-Kr-h1S154D - - - - - +
Flag-CBP - + + + + +
IP: a-Kr-h1
WB: a-Flag
Flag-Kr-h1
Flag-CBP
lysate
Flag-Kr-h1
Flag-CBP
Actin

Figure 6

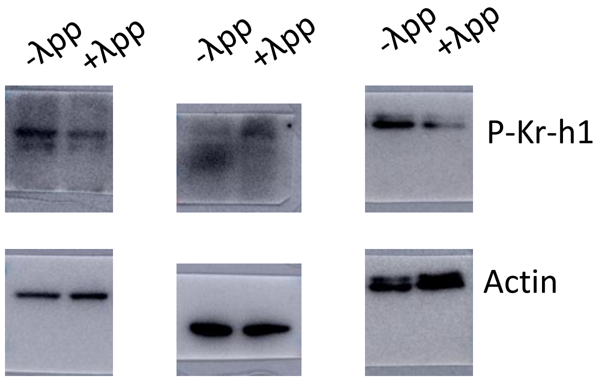

Figure S1

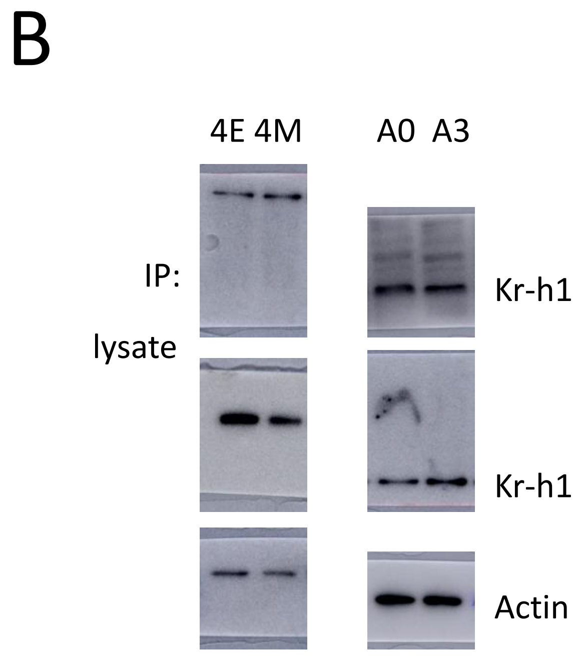
B
4E 4M
A0 A3
IP:
lysate
Kr-h1
Kr-h1
Actin

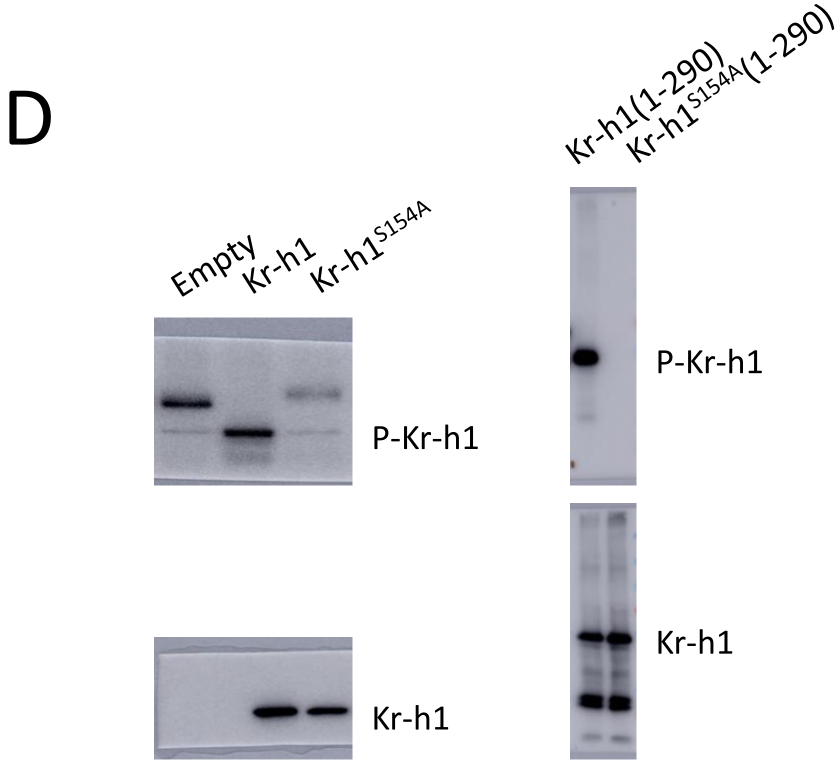
D
Empty
Kr-h1
Kr-h1^{S154A}
P-Kr-h1
Kr-h1
Kr-h1(1-290)
Kr-h1^{S154A}(1-290)
P-Kr-h1
Kr-h1

Figure S2

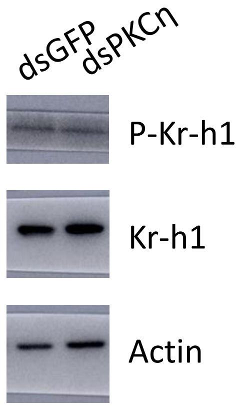

Figure S5

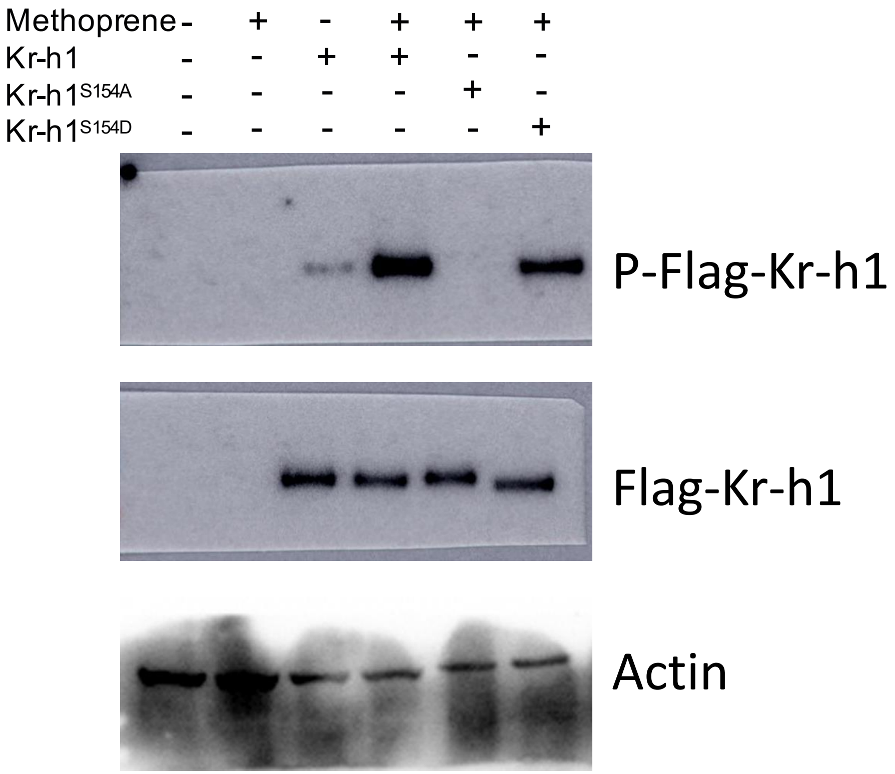
Methoprene - + - + + +
Kr-h1 - - + + - -
Kr-h1S154A - - - - + -
Kr-h1S154D - - - - - +
P-Flag-Kr-h1
Flag-Kr-h1
Actin
